# Effects of Amyloid Beta Peptide on Neurovascular Cells

**DOI:** 10.5195/cajgh.2012.4

**Published:** 2013-02-21

**Authors:** Sholpan Askarova, Andrey Tsoy, Tamara Shalakhmetova, James C-M Lee

**Affiliations:** 1 Nazarbayev University, Center for Life Sciences, Astana, Kazakhstan; 2 Al-Farabi Kazakh National University, Almaty, Kazakhstan; 3 Department of Biological Engineering, University of Missouri

**Keywords:** amyloid-beta petide, Alzheimer’s disease

## Abstract

Alzheimer’s disease (AD) is a chronic neurodegenerative disorder, which is characterized by the accumulation of amyloid plaques and neurofibrillary tangles in specific regions of the brain, accompanied by impairment of the neurons, and progressive deterioration of cognition and memory of affected individuals. Although the cause and progression of AD are still not well understood, the amyloid hypothesis is dominant and widely accepted. According to this hypothesis, an increased deposition of amyloid-β peptide (Aβ) in the brain is the main cause of the AD’s onset and progression. There is increasing body of evidence that blood-brain barrier (BBB) dysfunction plays an important role in the development of AD, and may even precede neuron degeneration in AD brain. In the early stage of AD, microvasculature deficiencies, inflammatory reactions, surrounding the cerebral vasculature and endothelial dysfunctions are commonly observed. Continuous neurovascular degeneration and accumulation of Aβ on blood vessels resulting in cerebral amyloid angiopathy is associated with further progression of the disease and cognitive decline. However, little is known about molecular mechanisms that underlie Aβ induced damage of neurovascular cells. In this regards, this review is aimed to address how Aβ impacts the cerebral endothelium. Understanding the cellular pathways triggered by Aβ leading to alterations in cerebral endothelial cells structure and functions would provide insights into the mechanism of BBB dysfunction and inflammatory processes in Alzheimer’s, and may offer new approaches for prevention and treatment strategies for AD.

## Introduction

Alzheimer’s disease is a chronic neurodegenerative disorder, which affects approximately 10% of the population at age 65 and 40% of people over the age 80. AD is characterized by the accumulation of amyloid plaques and neurofibrillary tangles accompanied by impairment of the neurons in specific regions of the brain. In particular, large neurons in the neurocortex, the entorhinal area, hippocampus, amygdale, nucleus basalis, anterior thalamus, and several brain stem monoaminergic nuclei are affected. In damaged regions, the neurons exhibit multiple abnormalities of cell structure and function, reduction in the level of synaptic proteins, and, finally, they die.^1^ The neuronal loss in AD brains is accompanied by progressive deterioration of cognition and memory of affected individuals.

Although the cause and progression of AD are still not well understood, the amyloid hypothesis is dominant and widely accepted.^2^ According to this hypothesis, an increased deposition of amyloid-β peptide, the main constituent of senile plagues, is the main cause of neuronal dysfunction and death in AD. The rest of the disease process, including formation of neurofibrillary tangles containing tau protein, is proposed to result from an imbalance between Aβ production and Aβ clearance. Aβ is derived from amyloidogenic cleavage of membrane bound amyloid precursor protein (APP) by β- and γ-secretase.^3^ Amyloidogenic processing of the APP leads to the production of Aβ peptides of different length, of which the Aβ^1^–^40^ is the major species and the Aβ^1^–^42^ is the most fibrillogenic and predominant component in AD plaques.^4^

There is major evidence which supports Amyloid Cascade Hypothesis. The first comes from the link between AD and Down’s Syndrome. The APP gene is localized on chromosome 21, and people with Down Syndrome (trisomy 21) who, thus, have an extra gene copy almost invariably develop AD-like neuropathology by the age of 40. Secondly, inherited mutations in the APP and presenilin genes (presenilin constitutes the catalytic site of the γ-secretase) cause early and aggressive forms of AD. And thirdly, transgenic mice with mutant form of human APP develop amyloid fibrillar plaques and Alzhemer’s like brain pathology.^2^

Recent reports have suggested that the soluble oligomeric form of the peptide is the most toxic and responsible for the disruption of synaptic plasticity, neuronal death and decline of cognitive function.^5^,^6^ Although precise mechanism of Aβ oligomers neurotoxic effects remains unclear, *in-vivo* and *in-vitro* studies have demonstrated that Aβ oligomers: a) induce apoptosis; b) initiate oxidative stress and free-radical degeneration in neuronal cells; c) disrupt calcium homeostasis and long-term potentiation; d) cause neurodegeneration by forming large, voltage independent, and nonselective ion channels.^7^,^8^

However, for the most cases of late-onset sporadic non-inherited AD (~95%), the reasons of increased Aβ accumulation in brains remain unknown. In this regard, current theories imply that AD is mainly caused by vascular risk factors, and that vascular derived pathology is responsible for initiation and/or progression of AD.^9^–^11^ Recent studies provided significant data supporting the notion that the pathophysiology of blood brain barrier (BBB) and imbalanced interaction between cerebral endothelial cells (CECs), glial cells and neurons may trigger the progressive destruction of cortical neurons in AD.^10^,^12^–^21^

## 1. Blood-Brain Barrier Disorder in AD

**Fig. 1:**
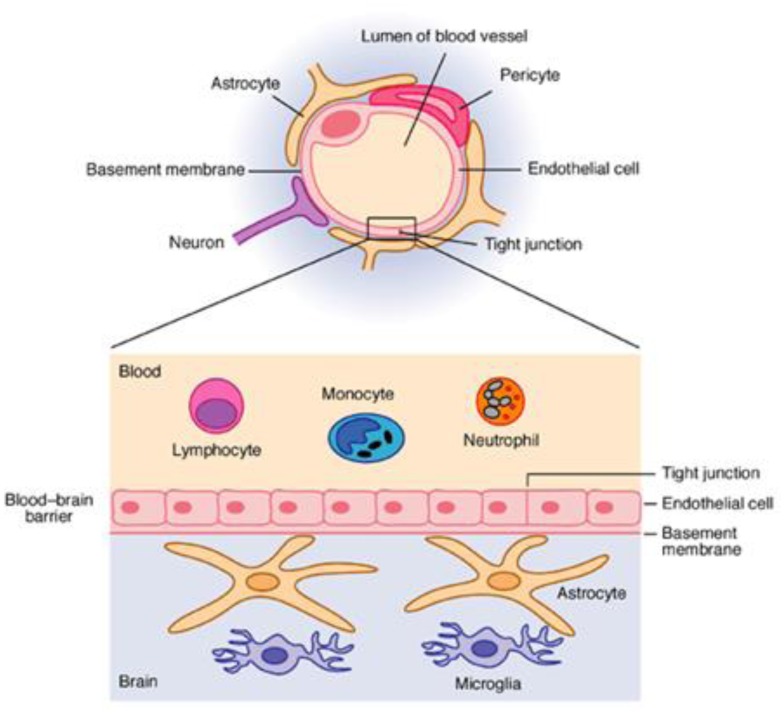
The Blood-Brain Barrier. (Adapted from Expert Reviews in Molecular Medicine, 2003 Cambridge University Press)

The homeostasis of the Central Nervous System (CNS) is maintained by the BBB, which separates the brain from the circulating bloodstream. The BBB is formed by a complex cellular system consisting of CECs, astrocytes, pericytes, perivascular macrophages, and a basement membrane (Fig 1.).^22^

CECs layer is a major component of the BBB which is comprised of high-density cells connected by tight junctions. CECs have a little number of endothelial pores, rich in mitochondria, and have a very low content of the pinocytic vesicles. The biomechanical properties of the CECs are critical to the regulation of many cellular functions, such as adhesion, signaling and morphology, and play a vital role for the maintenance of the BBB permeability, and brain parenchyma homeostasis. Astrocytes, the most frequent cells of the brain, also play an important role in maintaining BBB function. Their end feet tightly connected to the CECs influencing cerebrovascular tone and the barrier properties of endothelium.^20^ Pericytes are characterized as contractile cells that surround the brain capillaries. Pericytes play an important role in maintaining the stability of microvessels and modulation of Cerebral Blood Flow (CBF). Sporadic microglia can also be found in the surrounding pericapillary area in normal brain.^16^

There is increasing body of evidence that BBB dysfunction plays an important role in the development and progression of AD.^14^–^17^,^23^,^24^ Vascular disorders like atherosclerosis, ischemia, hypertension, and stroke are among the risk factors for AD.^18^,^20^,^21^ In the early stage of AD, microvasculature deficiencies, inflammatory reactions, surrounding the cerebral vasculature and endothelial dysfunctions are commonly observed.^25^ The increased number of perivascular macrophages and hypertrophy of astrocytes and microglia is commonly observed in AD brain sections.^26^ Numerous observations have indicated decreased cerebral blood flow, reduced total microvascular density, and low immunoreactivity of endothelial markers CD34 and CD3 in AD brains.^27^–^32^ Light and electron microscopy studies have demonstrated decreased mitochondrial and increased pinocytotic vesicles content, swelling and degeneration of endothelial cells.^33^,^34^

*In vitro*, amyloid beta peptide has been shown to induce significant dysfunctions in the CECs. Specifically, Aβ suppressed CECs proliferation and migration, affected tube formation in the human brain endothelial cells (HBEC), induced endothelial autophagy through the dissociation of ERK and AKT signaling and intracellular regulation of class 3 phosphatidylinositol 3-kinase.^35^,^36^ Physiological concentrations of soluble Aβ (10^−9^ – 10^−6^ M) induced dose-dependent reduction of NO production, decreased sensitivity of neurovasculature to an endothelium dependent vasodilator acetylcholine, increased cellular calcium level, initiated albumin transfer across EC monolayer and impaired EC glucose uptake.^24^,^37^–^39^ Higher concentrations of Aβ have been demonstrated to induce mitochondria dysfunction, nuclear chromatin condensation, DNA fragmentation, and significant cerebral endothelial cell death.^37^,^38^,^40^ Continuous neurovascular degeneration and accumulation of Aβ on blood vessels resulting in cerebral amyloid angiopathy is associated with further progression of the disease and cognitive decline^14^,^15^,^17^,^41^,^42^

## 2. Oxidative Stress, Inflammation, and Downstream Cell Signaling Pathways in AD

There is increasing evidence that oxidative stress is a main mechanism leading to cerebrovascular dysfunction in AD. Several studies of transgenic mice over expressing APP have demonstrated oxidative damage of CECs, up regulation of superoxide dismutase (SOD) around brain micro vessels, and significant impairment of the cerebrovascular functions.^43^–^45^ At the same time, endothelial dysfunctions were not observed in mice over expressing both APP and SOD-1 or in a case when SOD was directly applied to the cerebral cortex of the APP mice.^44^ *In vitro*, treatment of CECs with Aβ increased free radical production and this effect was attenuated by free radical scavengers.^43^,^46^

The oxidative stress initiates a cascade of redox reactions which trigger apoptosis. Several studies have indicated that Aβ-induced CECs death had an apoptotic nature and was a result of the mitochondria dysfunction, activation of a caspase upstream, and proapoptotic proteins release^38^,^40^,^47^,^48^ Aβ induced oxidative stress also triggers downstream kinase cascades leading to neurovascular inflammation.^49^,^50^ Study of the microvessels isolated from the AD patients brains have revealed significantly higher levels of interleukin-1β (IL-1β), IL-6, tumor necrosis factor α (TNF-α), microvessel-associated monocyte chemoattractant protein (MCP-1) and IL-1βs.^49^ *In-vitro*, the exposure of HBEC to Aβ induced induction of CD40 (a member of TNF receptor family), secretion of interferon-γ (IFN-γ) and IL-1β, expression of of IFN-γ receptor (IFN-γR), and triggered inflammatory genes MCP*-1*, *GRO*, *IL-1β* and *IL-6* expression via JNK-AP1 signaling pathway.^50^–^52^

Aβ-induced oxidative stress in cerebral epithelium is associated with overproduction of reactive oxygen species (ROS).^20^,^53^–^55^ ROS can be generated by several enzymatic systems, but there is evidence that superoxide-producing enzyme NADPH oxidase A is major source of ROS in the brain blood vessels.^54^–^56^ In a model of AD, inhibition of NADPH oxidase has been found to abrogate Aβ induced ROS production and alteration of cerebrovascular functions.^54^ APP transgenic mice lacking the NADPH oxidase subunits gp91^phox^ or Nox2 did not develop oxidative stress, cerebrovascular dysfunction, and behavioral deficits.^54^,^55^

Recent studies have indicated that the receptor for advanced glycation endproducts (RAGE) is a binding site for Aβ.^57^–^62^ RAGE is a multiligand cell surface receptor which is normally expressed in brain endothelium and, at low levels, in microglia and neurons.^15^,^60^,^61^ However, in AD brains RAGE expression is increased by several-fold in cerebral endothelial cells, astrocytes, microglia, and neurons.^60^,^61^ ROS have been reported to be generated by NADPH oxidase through the RAGE in endothelial cells.^62^,^63^ Inhibition studies have indicated that anti-RAGE IgG significantly suppressed oxidative stress and inflammation induced by Aβ in vascular cells and neurons.^57^ RAGE binding to Aβ has been also demonstrated to regulate Aβ transport across BBB, upregulate pro-inflammatory cytokines and adhesion molecules in CECs, and contribute to the transport of Aβ from the cell surface into the intracellular space in cortical neurons^61^,^64^,^65^

Aβ-induced cytotoxic effects are also associated with the activation of MAPK/ERK1/2 cascade and that activated ERKs (extracellular-signalregulated kinases) is the central target of RAGE.^62^,^66^–^72^ The ERKs are widely expressed protein kinases, part of a signal transduction system, through which extracellular stimuli are transduced. Activation of the ERKs occurs in response to growth factor stimulation, cytokines, virus infection, transforming agents, carcinogens, and after the activation of high-affinity IgG receptors.^71^ ERKs have been implicated in diverse cellular responses such as mitogenesis, differentiation, inflammation and cytotoxicity, and the overproduction of this enzyme is involved in many neurodegenerative diseases, including AD.^67^,^73^,^74^ Thus, NADPH oxidase, ERKs and RAGE have been suggested to be important therapeutic targets in AD.

## 3. Permeability of Cerebral Endothelium in AD

In the AD, an increased deposition of Aβ in the cerebral vasculature has been found to correlate with accumulation of monocytes in the vessel walls and of activated microglia cells in the adjacent parenchyma.^75^–^77^ Since peripheral monocytes can migrate across the BBB and differentiate into microglia,^78^ which, in turn, drives the disease development towards exacerbation of the oxidative and inflammatory conditions characteristic of the AD brain, several research groups have attempted to demonstrate the direct effect of Aβ on endothelial functions leading to enhanced transmigration of monocytes.

*In-vitro* studies have shown that soluble Aβ interactions with RAGE and platelet-endothelial cell adhesion molecule-1 (PECAM-1) at the apical surface and basolateral sides of monolayer of brain endothelial cells increased transendothelial migration of monocytic cells.^79^–^81^ Based on the observation that the permeability of the monolayer toward dextran and inulin in the presence or absence of Aβ^42^ remained unaltered,^79^ it has been concluded that enhanced transmigration of monocytes induced by Aβ is not only due to nonspecific disruption of the barrier properties of the endothelial layer, but also is a consequence of Aβ induced expression of the chemokines and adhesion molecules. Since primary capture of the monocytes to endothelium and rolling are mediated by tethering on selectins and selectin ligands,^82^–^84^ the expression of adhesion molecules, mechanical properties of the membranes (fluidity, elasticity) and membrane-cytoskeleton interactions are critical for transmigration.^85^–^90^ Atomic force microscopy and quantitative immunofluorescence microscopy studies have demonstrated that Aβ oligomers induced P-selectin expression, increased cell stiffness, decreased the apparent rupture force of selectin-ligand bonding due to dissociation of adhesion between the cytoskeleton and the bilayer membrane, and, thus, increased probability of adhesion. 91

The presence of the tight junctions of high electrical resistance and close cell-cell contact are important biomechanical factors maintaining brain homeostasis and BBB impermeability. Tight junction is a complex of transmembrane proteins (occluding, claudins, junctional molecule-1) and submembrane molecules connected to actin network. In fact, the structure and functions of the tight junctions are strongly affected in the cerebrovascular cells of AD patients.^92^ In an animal model of AD, a cholesterol-enriched diet down-regulated the expression of the occluding and ZO-1, which was strongly correlated with the elevated level of the BBB leakage.^93^ *In vitro*, treatment of primary rat CECs with Aβ^1^–^42^ for 3 days altered expression of occluding and claudin-1, caused relocation of plasma membrane subunits of claudin-5 and ZO-2 to the cytoplasm. At the same time, the cytoplasmic ZO-1 and ZO-2 where evenly distributed along the plasma membrane at the points of the cell-cell contacts.^94^ Apolipoprotein E4 (apoE4), a major risk factor for AD, has been shown to be involved in tight junction alteration as well.^95^ It has been shown that mice deficient in apoE have expressed BBB leakage. *In vitro* study has demonstrated that the barrier functions of tight junctions was impaired when the CECs were reconstituted with primary astrocytes from apoE4-knock-in mice. In particular, the phosphorylation of occludin and the activation of protein kinase C (PKC)η in CECs were attenuated.

These findings suggest that the effects of Aβ on actin and tight junction protein complexes, as well as vascular risk factors cause the alteration of endothelial layer integrity and contribute to the enhanced transmigration of monocytes across the BBB. Thus, studying the Aβ-mediated alterations in endothelial adhesion and BBB permeability would provide insights into the mechanism of BBB dysfunction and may provide information for developing new targeted drug delivery vehicles^96^ for the AD brain.

## Conclusion

Chronic neurovascular dysfunctions and degeneration of endothelium are observed in the all stages of AD. Numerous in vivo and in vitro studies have demonstrated that vascular deposition of amyloid beta peptide induces oxidative stress in cerebral vasculature, triggers inflammatory processes and apoptosis, promotes expression of adhesion molecules, affects tight junctions, changes mechanical properties of the CECs membranes, and enhances transmigration of immune cells across BBB. Continuous degeneration of CECs impairs BBB permeability and leads to leakage of blood cells, plasma components and neurotoxic substances into the brain parenchyma. Breakdown of blood brain barrier functions drives the disease development towards exacerbation of the oxidative and inflammatory conditions characteristic of the AD brain and contributes to further progression of the disease. Understanding the precise molecular mechanisms underlying Aβ-mediated oxidative stress in CECs, the effects of Aβ^42^ on the BBB adhesion and permeability should prove to provide new insights into the development of preventive and treatment strategies for AD.

## References

[1] Kandel ER, Schwartz JH, Jessel TM (2000). Principles of Neural Science.

[2] Hardy J, Selkoe DJ (2002). The Amyloid Hypothesis of Alzheimer’s Disease: Progress and Problems on the Road to Therapeutics. Science.

[3] Vassar R (2004). BACE1: the beta-secretase enzyme in Alzheimer’s disease. J Mol Neurosci.

[4] Bernstein SL, Wyttenbach T, Baumketner A, Shea J-E, Bitan G, Teplow DB (2005). Amyloid OI-Protein: вҔ‰ Monomer Structure and Early Aggregation States of AOI42 and Its Pro19 Alloform. Journal of the American Chemical Society.

[5] Walsh DM, Selkoe DJ (2007). Aβ Oligomers – a decade of discovery. Journal of Neurochemistry.

[6] Dahlgren KN, Manelli AM, Stine WB, Baker LK, Krafft GA, LaDu MJ (2002). Oligomeric and Fibrillar Species of Amyloid-OI Peptides Differentially Affect Neuronal Viability. Journal of Biological Chemistry.

[7] Resende R, Ferreiro E, Pereira C, Resende de Oliveira C (2008). Neurotoxic effect of oligomeric and fibrillar species of amyloid-beta peptide 1–42: Involvement of endoplasmic reticulum calcium release in oligomer-induced cell death. Neuroscience.

[8] Cleary JP, Walsh DM, Hofmeister JJ, Shankar GM, Kuskowski MA, Selkoe DJ (2005). Natural oligomers of the amyloid-[beta] protein specifically disrupt cognitive function. Nat Neurosci.

[9] Zlokovic BV (2011). Neurovascular pathways to neurodegeneration in Alzheimer’s disease and other disorders. Nat Rev Neurosci.

[10] Stanimirovic DB, Friedman A (2011). Pathophysiology of the neurovascular unit: disease cause or consequence[quest]. J Cereb Blood Flow Metab.

[11] Iadecola C (2010). The overlap between neurodegenerative and vascular factors in the pathogenesis of dementia. Acta Neuropathol.

[12] Liu R, Zhang T-t, Wu C-x, Lan X, Du G-h (2011). Targeting the neurovascular unit: Development of a new model and consideration for novel strategy for Alzheimer’s disease. Brain Research Bulletin.

[13] Salmina A, Inzhutova A, Malinovskaya N, Petrova M (2010). Endothelial dysfunction and repair in Alzheimer-type neurodegeneration: neuronal and glial control. J Alzheimers Dis.

[14] Ruitenberg A, den Heijer T, Bakker SL, van Swieten JC, Koudstaal PJ, Hofman A (2005). Cerebral hypoperfusion and clinical onset of dementia: the Rotterdam Study. Ann Neurol.

[15] Zlokovic BV (2008). New therapeutic targets in the neurovascular pathway in Alzheimer’s disease. Neurotherapeutics.

[16] Bell R, Zlokovic B (2009). Neurovascular mechanisms and blood–brain barrier disorder in Alzheimer’s disease. Acta Neuropathologica.

[17] Iadecola C (2003). Cerebrovascular effects of amyloid-beta peptides: mechanisms and implications for Alzheimer’s dementia. Cell Mol Neurobiol.

[18] de la Torre JC (2006). How do heart disease and stroke become risk factors for Alzheimer’s disease?. Neurological Research.

[19] Deane R, Zlokovic BV (2007). Role of the Blood-Brain Barrier in the pathogenesis of Alzheimers disease. Current Alzheimer Research.

[20] Girouard H, Iadecola C (2006). Neurovascular coupling in the normal brain and in hypertension, stroke, and Alzheimer disease. J Appl Physiol.

[21] Hofman A, Ott A, Breteler MMB, Bots ML, Slooter AJC, van Harskamp F (1997). Atherosclerosis, apolipoprotein E, and prevalence of dementia and Alzheimer’s disease in the Rotterdam Study. The Lancet.

[22] Bradbury MW (1985). The blood-brain barrier. Transport across the cerebral endothelium. Circ Res.

[23] Scheibel A, Duong T, Jacobs R (1989). Alzheimer’s disease as a capillary dementia. Ann Med.

[24] De la Torre JC (2004). Is Alzheimer’s disease a neurodegenerative or a vascular disorder? Data, dogma, and dialectics. The Lancet Neurology.

[25] Borroni B, Akkawi N, Martini G, Colciaghi F, Prometti P, Rozzini L (2002). Microvascular damage and platelet abnormalities in early Alzheimer’s disease. Journal of the Neurological Sciences.

[26] Farkas E, Luiten PGM (2001). Cerebral microvascular pathology in aging and Alzheimer’s disease. Progress in Neurobiology.

[27] Luc B, Patrick RHOF, AndrЙ D (1997). Brain Microvascular Changes in Alzheimer’s Disease and Other Dementias^a^. Annals of the New York Academy of Sciences.

[28] Berzin TM, Zipser BD, Rafii MS, Kuo--Leblanc V, Yancopoulos GD, Glass DJ (2000). Agrin and microvascular damage in Alzheimer’s disease. Neurobiology of Aging.

[29] Bailey TL, Rivara CB, Rocher AB, Hof PR (2004). The nature and effects of cortical microvascular pathology in aging and Alzheimer’s disease. Neurological Research.

[30] Kalaria RN, Pax AB (1995). Increased collagen content of cerebral microvessels in Alzheimer’s disease. Brain Research.

[31] Ervin JF, Pannell C, Szymanski M, Welsh-Bohmer K, Schmechel DE, Hulette CM (2004). Vascular Smooth Muscle Actin Is Reduced in Alzheimer Disease Brain: A Quantitative Analysis. Journal of Neuropathology & Experimental Neurology.

[32] Kalaria RNHP (1995). Differential degeneration of the cerebral microvasculature in Alzheimer’s disease. Neuroreport.

[33] Claudio L (1996). Ultrastructural features of the blood-brain barrier in biopsy tissue from Alzheimer’s disease patients. Acta Neuropathologica.

[34] Aliev G, Seyidova D, Lamb BT, Obrenovich ME, Siedlak SL, Vinters HV (2003). Mitochondria and vascular lesions as a central target for the development of Alzheimer’s disease and Alzheimer disease-like pathology in transgenic mice. Neurological Research.

[35] Magrane J, Christensen RA, Rosen KM, Veereshwarayya V, Querfurth HW (2006). Dissociation of ERK and Akt signaling in endothelial cell angiogenic responses to [beta]-amyloid. Experimental Cell Research.

[36] Hayashi S-i, Sato N, Yamamoto A, Ikegame Y, Nakashima S, Ogihara T (2009). Alzheimer Disease-Associated Peptide, Amyloid {beta}40, Inhibits Vascular Regeneration With Induction of Endothelial Autophagy. Arterioscler Thromb Vasc Biol.

[37] Price JM, Chi X, Hellermann G, Sutton ET (2001). Physiological levels of -amyloid induce cerebral vessel dysfunction and reduce endothelial nitric oxide production. Neurological Research.

[38] Emmanuelle MB, Michal T, Robert JM, Bernhard H (1997). Amyloid β-Peptide Induces Cell Monolayer Albumin Permeability, Impairs Glucose Transport, and Induces Apoptosis in Vascular Endothelial Cells. Journal of Neurochemistry.

[39] Bhatia R, Lin HAI, Lal R (2000). Fresh and globular amyloid {beta} protein (1–42) induces rapid cellular degeneration: evidence for A{beta}P channel-mediated cellular toxicity. FASEB J.

[40] Xu J, Chen S, Ku G, Ahmed SH, Xu J, Chen H (2001). Amyloid beta Peptide-Induced Cerebral Endothelial Cell Death Involves Mitochondrial Dysfunction and Caspase Activation. J Cereb Blood Flow Metab.

[41] Selkoe DJ, SD (2003). Alzheimer’s disease: molecular understanding predicts amyloid-based therapeutics. Annu Rev Pharmacol Toxicol.

[42] Kalaria RN (2006). Cerebrovascular Degeneration Is Related to Amyloid-β Protein Deposition in Alzheimer’s Disease. Annals of the New York Academy of Sciences.

[43] Park LAJ, Forster C, Kazama K, Carlson GA, Iadecola C (2004). Abeta-induced vascular oxidative stress and attenuation of functional hyperemia in mouse somatosensory cortex. Joyrnal of Cerebral Blood Flow Metabolism.

[44] Iadecola CZF, Niwa K, Eckman C, Turner SK, Fischer E, Younkin S, Borchelt DR, Hsiao KK, Carlson GA (1999). SOD1 rescues cerebral endothelial dysfunction in mice overexpressing amyloid precursor protein. Nature Neuroscience.

[45] Tong X-K, Nicolakakis N, Kocharyan A, Hamel E (2005). Vascular Remodeling versus Amyloid-beta-Induced Oxidative Stress in the Cerebrovascular Dysfunctions Associated with Alzheimer’s Disease. J Neurosci.

[46] Abramov AY, Duchen MR (2005). The role of an astrocytic NADPH oxidase in the neurotoxicity of amyloid beta peptides. Philosophical Transactions of the Royal Society B: Biological Sciences.

[47] Yin KJ, Lee JM, Chen SD, Xu J, Hsu CY (2002). Amyloid-beta Induces Smac Release via AP-1/Bim Activation in Cerebral Endothelial Cells. J Neurosci.

[48] Hsu M-J, Hsu CY, Chen B-C, Chen M-C, Ou G, Lin C-H (2007). Apoptosis Signal-Regulating Kinase 1 in Amyloid {beta} Peptide-Induced Cerebral Endothelial Cell Apoptosis. J Neurosci.

[49] Grammas P, Ovase R (2001). Inflammatory factors are elevated in brain microvessels in Alzheimer’s disease. Neurobiology of Aging.

[50] Vukic V, Callaghan D, Walker D, Lue L-F, Liu QY, Couraud P-O (2009). Expression of inflammatory genes induced by beta-amyloid peptides in human brain endothelial cells and in Alzheimer’s brain is mediated by the JNK-AP1 signaling pathway. Neurobiology of Disease.

[51] Tan J, Town T, Suo Z, Wu Y, Song S, Kundtz A (1999). Induction of CD40 on human endothelial cells by Alzheimer’s [beta]-amyloid peptides. Brain Research Bulletin.

[52] Suo Z, Tan J, Placzek A, Crawford F, Fang C, Mullan M (1998). Alzheimer’s [beta]-amyloid peptides induce inflammatory cascade in human vascular cells: the roles of cytokines and CD40. Brain Research.

[53] Callaghan D, Bai J, Huang A, Vukic V, Xiong H, Jones A (2008). P4–182: Inhibition of ABCG2 transport function by amyloid-beta peptide augments cellular oxidative stress and inflammatory gene expression in cells. Alzheimer’s and Dementia.

[54] Park L, Anrather J, Zhou P, Frys K, Pitstick R, Younkin S (2005). NADPH Oxidase-Derived Reactive Oxygen Species Mediate the Cerebrovascular Dysfunction Induced by the Amyloid {beta} Peptide. J Neurosci.

[55] Park L, Zhou P, Pitstick R, Capone C, Anrather J, Norris EH (2008). Nox2-derived radicals contribute to neurovascular and behavioral dysfunction in mice overexpressing the amyloid precursor protein. Proceedings of the National Academy of Sciences.

[56] Cai H, Griendling KK, Harrison DG (2003). The vascular NAD(P)H oxidases as therapeutic targets in cardiovascular diseases. Trends in Pharmacological Sciences.

[57] Yan SD, Chen X, Fu J, Chen M, Zhu H, Roher A (1996). RAGE and amyloid-[beta] peptide neurotoxicity in Alzheimer’s disease. Nature.

[58] Arancio O, Zhang HP, Chen X, Lin C, Trinchese F, Puzzo D (2004). RAGE potentiates A[beta]-induced perturbation of neuronal function in transgenic mice. EMBO J.

[59] Chaney MO, Stine WB, Kokjohn TA, Kuo Y-M, Esh C, Rahman A (2005). RAGE and amyloid beta interactions: Atomic force microscopy and molecular modeling. Biochimica et Biophysica Acta (BBA) - Molecular Basis of Disease.

[60] Sasaki N, Toki S, Chowei H, Saito T, Nakano N, Hayashi Y (2001). Immunohistochemical distribution of the receptor for advanced glycation end products in neurons and astrocytes in Alzheimer’s disease. Brain Research.

[61] Lue L-F, Walker DG, Brachova L, Beach TG, Rogers J, Schmidt AM (2001). Involvement of Microglial Receptor for Advanced Glycation Endproducts (RAGE) in Alzheimer’s Disease: Identification of a Cellular Activation Mechanism. Experimental Neurology.

[62] Askarova S, Yang X, Sheng W, Sun GY, Lee JC-M (2011). Role of Aβ-Receptor for advanced endproducts in oxidativestress and cytosolic phospholipase A2 activation in astrocytes and cerebral endothelial cells. Neuroscience.

[63] Wautier M-P, Chappey O, Corda S, Stern DM, Schmidt AM, Wautier J-L (2001). Activation of NADPH oxidase by AGE links oxidant stress to altered gene expression via RAGE. Am J Physiol Endocrinol Metab.

[64] Giri R, Shen Y, Stins M, Du Yan S, Schmidt AM, Stern D (2000). Beta-amyloid-induced migration of monocytes across human brain endothelial cells involves RAGE and PECAM-1. Am J Physiol Cell Physiol.

[65] Takuma K, Fang F, Zhang W, Yan S, Fukuzaki E, Du H (2009). RAGE-mediated signaling contributes to intraneuronal transport of amyloid-OI and neuronal dysfunction. Proceedings of the National Academy of Sciences.

[66] Zhu D, Lai Y, Shelat PB, Hu C, Sun GY, Lee JCM (2006). Phospholipases A2 Mediate Amyloid-beta Peptide-Induced Mitochondrial Dysfunction. J Neurosci.

[67] Stephenson DT, Lemere CA, Selkoe DJ, Clemens JA (1996). Cytosolic phospholipase A2 (cPLA2) immunoreactivity is elevated in Alzheimer’s disease brain. Neurobiol Dis.

[68] Moses GS, Jensen MD, Lue LF, Walker DG, Sun AY, Simonyi A (2006). Secretory PLA2-IIA: a new inflammatory factor for Alzheimer’s disease. J Neuroinflammation.

[69] Dineley KT, Westerman M, Bui D, Bell K, Ashe KH, Sweatt JD (2001). {beta}-Amyloid Activates the Mitogen-Activated Protein Kinase Cascade via Hippocampal {alpha}7 Nicotinic Acetylcholine Receptors: In Vitro and In Vivo Mechanisms Related to Alzheimer’s Disease. J Neurosci.

[70] Young KF, Pasternak SH, Rylett RJ (2009). Oligomeric aggregates of amyloid [beta] peptide 1–42 activate ERK/MAPK in SH-SY5Y cells via the [alpha]7 nicotinic receptor. Neurochemistry International.

[71] McDonald DR, Bamberger ME, Combs CK, Landreth GE (1998). beta -Amyloid Fibrils Activate Parallel Mitogen-Activated Protein Kinase Pathways in Microglia and THP1 Monocytes. J Neurosci.

[72] Shelat PB, Chalimoniuk M, Wang J-H, Strosznajder JB, Lee JC, Sun AY (2008). Amyloid beta peptide and NMDA induce ROS from NADPH oxidase and AA release from cytosolic phospholipase A^2^ in cortical neurons. Journal of Neurochemistry.

[73] Stephenson D, Rash K, Smalstig B, Roberts E, Johnstone E, Sharp J (1999). Cytosolic phospholipase A2 is induced in reactive glia following different forms of neurodegeneration. Glia.

[74] Sun GY, Horrocks LA, Farooqui AA (2007). The roles of NADPH oxidase and phospholipases A^2^ in oxidative and inflammatory responses in neurodegenerative diseases. Journal of Neurochemistry.

[75] Maat-Schieman MLvDS, Rozemuller AJ, Haan J, Roos RA (1997). Association of vascular amyloid beta and cells of the mononuclear phagocyte system in hereditary cerebral hemorrhage with amyloidosis (Dutch) and Alzheimer disease. J Neuropathol Exp Neurol.

[76] Uchihara TAH, Kondo H, Ikeda K (1997). Activated microglial cells are colocalized with perivascular deposits of amyloid-beta protein in Alzheimer’s disease brain. Stroke.

[77] Selkoe DJ, Schenk D (2003). ALZHEIMER’S DISEASE: Molecular Understanding Predicts Amyloid-Based Therapeutics. Annual Review of Pharmacology and Toxicology.

[78] Mezey E, Chandross KJ, Harta G, Maki RA, McKercher SR (2000). Turning Blood into Brain: Cells Bearing Neuronal Antigens Generated in Vivo from Bone Marrow. Science.

[79] Giri R, Selvaraj S, Miller CA, Hofman F, Yan SD, Stern D (2002). Effect of endothelial cell polarity on beta -amyloid-induced migration of monocytes across normal and AD endothelium. Am J Physiol Cell Physiol.

[80] Reyes Barcelo A, Gonzalez-Velasquez F, Moss M (2009). Soluble aggregates of the amyloid-beta peptide are trapped by serum albumin to enhance amyloid-beta activation of endothelial cells. Journal of Biological Engineering.

[81] Gonzalez-Velasquez FJ, Kotarek JA, Moss MA (2008). Soluble aggregates of the amyloid-beta protein selectively stimulate permeability in human brain microvascular endothelial monolayers. J Neurochem.

[82] Frijns CJ, Kappelle LJ (2002). Inflammatory cell adhesion molecules in ischemic cerebrovascular disease. Stroke.

[83] Alon R, Chen S, Puri KD, Finger EB, Springer TA (1997). The kinetics of L-selectin tethers and the mechanics of selectin-mediated rolling. J Cell Biol.

[84] Alon R, Hammer DA, Springer TA (1995). Lifetime of the P-selectin-carbohydrate bond and its response to tensile force in hydrodynamic flow. Nature.

[85] Dembo M, Torney DC, Saxman K, Hammer D (1988). The reaction-limited kinetics of membrane-to-surface adhesion and detachment. Proc R Soc Lond B Biol Sci.

[86] Trache A, Trzeciakowski JP, Gardiner L, Sun Z, Muthuchamy M, Guo M (2005). Histamine effects on endothelial cell fibronectin interaction studied by atomic force microscopy. Biophys J.

[87] Sun M, Northup N, Marga F, Huber T, Byfield FJ, Levitan I (2007). The effect of cellular cholesterol on membranecytoskeleton adhesion. J Cell Sci.

[88] Sun M, Graham JS, Hegedьs B, Marga F, Zhang Y, Forgacs G (2005). Multiple Membrane Tethers Probed by Atomic Force Microscopy.

[89] Girdhar G, Shao J-Y (2004). Membrane Tether Extraction from Human Umbilical Vein Endothelial Cells and Its Implication in Leukocyte Rolling. Biophysical Journal.

[90] Girdhar G, Chen Y, Shao J-Y (2007). Double-Tether Extraction from Human Umbilical Vein and Dermal Microvascular Endothelial Cells. Biophysical Journal.

[91] Lee JCM, Askarova S, Sun Z, Sun GY, Meininger GA (2008). P4-293: Oligomeric amyloid-OI peptide on sialyl LewisX-selectin bonding at the cerebral endothelial cell surface. Alzheimer’s & dementia: the journal of the Alzheimer’s Association.

[92] Bednarczyk J, Lukasiuk K (2011). Tight junctions in neurological diseases. Acta Neurobiol Exp.

[93] Chen X, Gawryluk J, Wagener J, Ghribi O, Geiger J (2008). Caffeine blocks disruption of blood brain barrier in a rabbit model of Alzheimer’s disease. Journal of Neuroinflammation.

[94] Marco S, Skaper SD (2006). Amyloid [beta]-peptide1–42 alters tight junction protein distribution and expression in brain microvessel endothelial cells. Neuroscience Letters.

[95] Nishitsuji K, Hosono T, Nakamura T, Bu G, MM (2011). Apolipoprotein E regulates the integrity of tight junctions in an isoform-dependent manner in an in vitro blood-brain barrier model. J Biol Chem.

[96] Omolola Eniola A, Hammer DA (2005). In vitro characterization of leukocyte mimetic for targeting therapeutics to the endothelium using two receptors. Biomaterials.

